# Benchmarking freely available HLA typing algorithms across varying genes, coverages and typing resolutions

**DOI:** 10.3389/fimmu.2022.987655

**Published:** 2022-11-08

**Authors:** Nikolas Hallberg Thuesen, Michael Schantz Klausen, Shyam Gopalakrishnan, Thomas Trolle, Gabriel Renaud

**Affiliations:** ^1^ Evaxion Biotech, Copenhagen, Denmark; ^2^ Department of Health Technology, Section for Bioinformatics, Technical University of Denmark, Lyngby, Denmark; ^3^ Section for Hologenomics, Department of Biology, University of Copenhagen, Copenhagen, Denmark

**Keywords:** human leukycote antigen, next-generation sequencing (NGS), whole exome sequencing, depth of coverage, algorithm, benchmark, typing resolution

## Abstract

Identifying the specific human leukocyte antigen (HLA) allele combination of an individual is crucial in organ donation, risk assessment of autoimmune and infectious diseases and cancer immunotherapy. However, due to the high genetic polymorphism in this region, HLA typing requires specialized methods. We investigated the performance of five next-generation sequencing (NGS) based HLA typing tools with a non-restricted license namely HLA*LA, Optitype, HISAT-genotype, Kourami and STC-Seq. This evaluation was done for the five HLA loci, HLA-A, -B, -C, -DRB1 and -DQB1 using whole-exome sequencing (WES) samples from 829 individuals. The robustness of the tools to lower depth of coverage (DOC) was evaluated by subsampling and HLA typing 230 WES samples at DOC ranging from 1X to 100X. The HLA typing accuracy was measured across four typing resolutions. Among these, we present two clinically-relevant typing resolutions (P group and pseudo-sequence), which specifically focus on the peptide binding region. On average, across the five HLA loci examined, HLA*LA was found to have the highest typing accuracy. For the individual loci, HLA-A, -B and -C, Optitype’s typing accuracy was the highest and HLA*LA had the highest typing accuracy for HLA-DRB1 and -DQB1. The tools’ robustness to lower DOC data varied widely and further depended on the specific HLA locus. For all Class I loci, Optitype had a typing accuracy above 95% (according to the modification of the amino acids in the functionally relevant portion of the HLA molecule) at 50X, but increasing the DOC beyond even 100X could still improve the typing accuracy of HISAT-genotype, Kourami, and STC-seq across all five HLA loci as well as HLA*LA’s typing accuracy for HLA-DQB1. HLA typing is also used in studies of ancient DNA (aDNA), which is often based on sequencing data with lower quality and DOC. Interestingly, we found that Optitype’s typing accuracy is not notably impaired by short read length or by DNA damage, which is typical of aDNA, as long as the DOC is sufficiently high.

## 1 Introduction

**Figure 1:**
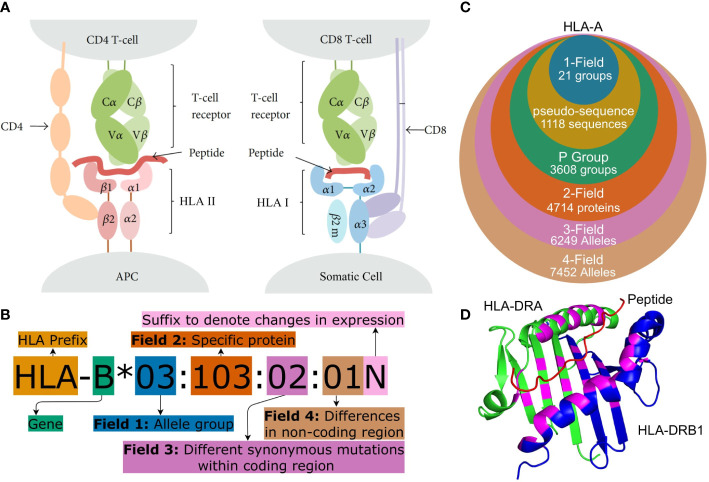
**(A)** Peptide presentation on APCs to (I) a CD4^+^ helper T-cell *via* an HLA Class II molecule and (II) to a CTL (CD8^+^ T cell) *via* an HLA Class I molecule. Both Class I and Class II molecules are heterodimers but for Class II, the ARD is made up of one domain from each monomer and therefore encoded by two different genes. Figure adapted from (1). **(B)** HLA nomenclature shown with full four field (8-digit) resolution. Adapted from http://hla.alleles.org/nomenclature/naming.html. **(C)** The number of HLA alleles varies greatly with each typing resolution. In this figure, “pseudo-sequence” refers to the amino acid residues directly involved with the binding of the peptide as shown in **(D)**. Each of the typing resolutions shown in this figure are subgroups of the higher typing resolutions, meaning that it is always possible to convert unambiguously from e.g. 2-field resolution to P group resolution. Note that null alleles are disregarded at P group and pseudo-sequence resolution, as these do not correspond to an expressed protein. The data is acquired from the IPD-IMGT/HLA database (2) release 3.48.0. **(D)** A binding pocket of an HLA Class II molecule (HLA-DR). The specific residues, which are directly involved in binding a peptide to the HLA molecule, are highlighted in purple. These purple residues make up the pseudo-sequence. DRA is shownin green and DRB1 in blue and a melanoma antigen in the binding pocket is shown in red. Protein data was obtained from the Protein Data Bank (3, 4) and the figure was made using PyMOL (5) panels **(A–D)** were crated using https://www.diagrams.net/.

Human leukocyte antigens (HLA) are a group of genes in the Major Histocompatibility Complex (MHC) region. They encode membrane-bound proteins involved with peptide presentation to T-cells and are central to the adaptive immune system. HLA Class I molecules are found on the surface of most somatic cells and present peptides, originating from proteins produced within the cell, to CD8^+^ cytotoxic T lymphocytes (CTLs), while HLA Class II molecules are found on antigen-presenting cells (APCs) and present exogenous peptides to CD4^+^ helper T-cells (see **Figure 1A**) (2, 6, 7).

Cells can present peptides to T-cells both from pathogens (non-self peptides), tumor mutations (neopeptides) and cells native to the body (self-peptides). T-cells are however generally able to recognise the difference between self and foreign antigens. This means that a cell displaying a non-self peptide, thereby indicating that it is infected by a virus or developing into a tumor cell, can trigger an immune response that a healthy cell would otherwise avoid (8, 9). The binding of a peptide to HLA and subsequently the recognition of this complex by a T-cell receptor (TCR) is highly specific, and a given HLA molecule will only bind and display a peptide if it matches the HLA molecule’s binding cleft, which is also known as the peptide-binding groove and as the antigen recognition domain (ARD) (10, 11). The MHC region is highly polymorphic and the most diverse sites are HLA Class I loci, HLA-A, -B and -C as well as the HLA Class II loci HLA-DRB1, -DQB1 and -DPB1. The IPD-IMGT/HLA database (Release 3.49.0) lists more than 31000 unique HLA allotype sequences and more than 19000 unique proteins for these six loci alone (2). HLA genes are, furthermore, co-dominantly expressed, giving an enormous amount of possible HLA profiles for an individual (12).

Due to the large number of alleles, the naming of specific HLA alleles follows a special convention as illustrated in **Figures 1B, C** (13, 14). HLA nomenclature is comprised of up to four fields and each additional field describes a specific allele with increasing precision.

The most important part of the HLA molecule is the ARD, which is encoded by exons 2 and 3 in Class I molecules and by exon 2 in HLA Class II molecules. The most important sequence differences between alleles are therefore the ones affecting the nucleotides in this region (13). Two official ARD-based HLA typing resolutions exist. G group resolution which clusters alleles with identical nucleotide sequences in ARD coding exons and P group resolution which groups alleles with identical ARD protein sequences. An overview of these can be found at^1^. A 2019 article (15) argued that mismatches outside the ARD are generally not important and that clinical decision-making therefore should focus on the ARD sequence except for common null alleles that are distinguished by variation outside the ARD. This recommendation is followed by using P group resolution and accounting for null alleles separately. Alleles can be further grouped based on the residues that are directly involved with the binding of the peptide to the HLA molecule (see **Figure 1D**). This grouping method is used in tools predicting peptide-HLA binding such as NetMHCpan (16).

HLA typing is the process of determining an individual’s specific HLA alleles. HLA typing is used widely since the peptide presentation is a crucial part of the adaptive immune system and depends on the specific HLA allele. Some examples include the study and prognosis of infectious diseases, autoimmune diseases and cancer, as well as the discovery of neoantigens in cancer treatment and for finding compatible donors for organ transplants (10, 17, 18).

HLA typing is significantly more difficult than traditional variant calling. This is mainly due to the extremely high degree of variation in the MHC region and a high degree of sequence homology between different HLA loci (12). Traditional HLA typing uses lab-based methods which can be slow and expensive. The rapid development of next-generation sequencing (NGS) has, however, resulted in large amounts of easily available sequencing data which can be used for HLA typing by employing recently developed computational tools (19–23).

NGS-based HLA typing tools can be divided roughly into two groups - those using *de novo* assembly-based methods and those which directly align to a reference sequence. The alignment-based methods either use a traditional linear reference or a graph-based reference/graph-based alignment algorithm (20, 24). The tools further differ on which HLA genes they can type and on the sequencing data, which they use for typing.

A 2019 review of NGS-based HLA typing noted the lack of systematic benchmarking of the many available HLA typing algorithms (24) and although several benchmarking studies have been published, there is no specific tool that consistently outperforms the others. Instead, the studies have shown that the optimal choice of HLA typing tool differs between sequencing data types and specific HLA loci (25–29). Some tools, such as Optitype (19) and Polysolver (30) only offer typing of Class I genes, while other tools have been developed specifically for use with high-coverage whole-genome sequencing (WGS) data (21).

New alleles are registered and named by a World Health Organisation committee and stored in the IPD-IMGT/HLA database. The database is continuously updated and errors are corrected but the database is not complete. New alleles are still being discovered and the full genomic sequence is not known for all registered alleles. Some entries are still missing the non-ARD coding exons and/or the introns (2).

The application of NGS-based HLA typing is not limited to presently living individuals but has also been used in studies of ancient genomes for example to find specific HLA alleles that increase susceptibility or protection to a specific disease. Sequencing of ancient DNA (aDNA) is often limited by a low depth of coverage (DOC), short DNA sequence length and chemical damage to the DNA (31). However, studies of aDNA have still used modern HLA typing tools such as Optitype (32) and an adaptation of HLAssign (33) to perform HLA typing on ancient individuals. Optitype is designed for general, non-enriched sequencing data (19) but has been used to type aDNA samples due to its apparent reliability for sequencing data with low DOC and/or read length. However, it has been noted that Optitype has not yet been tested or validated on aDNA and it is therefore also used along with non-automated, aDNA specialised pipelines such as the TARGT pipeline (34).

In this study, we present a comprehensive review of the performance of freely available HLA typing methods based on NGS. Specifically, the five HLA loci HLA-A, -B, -C, -DRB1 and -DQB1 are typed using whole-exome sequencing (WES) data. WES data is widely used in clinical settings as it is an affordable alternative to WGS while still providing a general genomic profile of a patient. This data is therefore often readily available for a patient (35, 36). This study is limited to these five HLA loci as the chosen reference dataset only contains sequence-based typing for those (see section 2.2). These loci are, however, also the most important and often the main focus of clinical HLA analyses (37).

This study demonstrates the first use of P group resolution and pseudo-sequence resolution in a benchmarking study of WES-based HLA typing. We find that HLA*LA had the highest typing accuracy across the five HLA loci, and that the typing resolution does not have an effect on which tool performed the best. We show that the impact of the DOC on the HLA typing accuracy depends heavily on both the tool and the HLA locus. A DOC of at least 100X is advisable for accurate typing of all five HLA genes - even for the best performing tools.

An understanding of the impact of the DOC on typing accuracy is crucial in HLA studies based on aDNA. We, therefore, expand upon the previous results by estimating Optitype’s performance on simulated aDNA that mimics aDNA samples in terms of DOC, read length and adding simulated chemical damage. Interestingly, we find that read length does not matter as much as the DOC however, Optitype requires a DOC between 10X and 20X to achieve a typing accuracy above 90% which is often prohibitively high for ancient DNA samples.

## 2 Materials and methods

### 2.1 Selection of HLA typing tools

There are numerous NGS-based HLA typing tools available. This study focused on freely available tools which ran on WES data and had shown promising results in previous benchmarking or proof of concept studies. This means that tools such as HLA-HD (38), Polysolver (30) and OncoHLA (39), which require some form of license, were not included in this study. The final selection of tools is listed in **Table 1**. STC-Seq was included as a reference to illustrate how a simpler algorithm, which is designed for HLA enriched data, performs on WES data with lower DOC. Optitype was downloaded from^2^. CBC 2.9.5 was used as ILP solver as it was found to be more stable than CPLEX 12.7 which often did not converge to a solution. Kourami was downloaded from^3^, HLA*LA was downloaded from^4^, HISAT-genotype was installed using its web-guide at^5^ and STC-Seq was downloaded from the BioCode website^6^.

**Table 1:** Details of the five HLA typing algorithms included in this project.

Tool	Version	Resolution	Approach	Reason for inclusion in this study	Known disadvantages
Kourami (21)	0.9.6	G group	Weighted graph structure from alignment of input reads aligned to reference sequences. Most probable graph path is the inferred type.	High typing accuracy in previous studies (39). Can detect and report new alleles, which are not included in a database.	Build for WGS based and is negatively affected by gaps in the sequencing data.
HLA*LA (23)	1.0.1	G group	Linear alignments projected on a population reference graph. Likelihood functions to infer the HLA type.	High typing accuracy in previous studies (39).	
HISAT-genotype (20)	1.3.2	4-field	Graph-based alignment (HISAT2) and an expectation maximisation algorithm.	High typing accuracy in previous studies (40). Able to detect novel alleles. Unique, as it does not include some form of linear alignment, but an extension of BWT for graphs.	
STC-Seq (22)	1.0	3-field	Dense chip-based probes that capture the coding regions of HLA. Linear alignment algorithm.	Offers a perspective by showing the performance of a simpler bioinformatics approach, as it is designed for HLA enriched data.	Not designed for general sequencing data.
Optitype (19)	1.3.3	2-field	Integer linear programming to find the allele combination that explains the highest number of reads.	High typing accuracy in previous studies (25, 26, 28)	

Each of the original articles describing the tools contains some sort of benchmarking study, demonstrating the capabilities of the tool. For more a more extensive outline of these five HLA typing tools, see **Supplementary Table S1**.

All tools in the analysis were run with default parameters and their own version of the IMGT database. This means, that potential advantages of more advanced features of some tools were not tested. Each tool was given 10 threads and as much memory as needed.

### 2.2 Benchmarking dataset

To evaluate the performance of the HLA typing tools, we used a reference dataset consisting of WES samples from 829 individuals taken from the 1000 genomes phase 3 dataset (41). The dataset contained samples from across the world with 340 being European, 187 African, 201 Ad Mixed American, and 101 East Asian. The DOC of the samples (measured over the targeted exons) ranged from 37X to 456X with the median being 86X.

819 of the 829 samples were typed by (42), who determined the HLA types by PCR amplification and Sanger sequencing followed by sequence comparison to the ARD coding exons (IPD-IMGT/HLA version 2.26.0). This method resulted in ambiguous typing results for some samples e.g NA12287 which had the HLA-B typing: 15:01/15:03 for the first allele and 15:01/15:26/15:12/15:19 for the second allele. The last 10 samples were typed by (43), who used a similar amplification and sequencing method but compared the sequencing data to both the ARD coding exons as well as exon 4 for Class I genes and exon 3 for HLA-DQB1. For these 10 samples, ambiguities were resolved using sequencing with sequence-specific primers and the typing was further validated using sequence-specific oligonucleotide probe hybridization.

All 829 samples were further investigated in a 2018 study (44) (using IPD-IMGT/HLA version 3.28) who found some disagreements between their own typing results and the findings from (42). These disagreements were manually investigated and some of the typing results by (42) were found to be typing errors.

In this study, we used the - occasionally ambiguous - typing results of (42) and (43), but updated the typing for the specific alleles in, which (44) had found to be mistyped. We further found that this dataset contained some alleles, whose names have changed since 2014. For both the gold standard dataset and for the predictions made by the HLA typing tools, these alleles were converted to their newest name. A full overview of all deleted/renamed alleles can be found at ^7^. This gold standard dataset is referred to as the 1000G dataset in the remainder of the paper.

For the majority of the individuals in the 1000G dataset, the HLA typing was only available in 2-field resolution and the tools could therefore only be evaluated at 2-field resolution or lower. In the cases, where the reference dataset contained ambiguous typing, predictions by the tools were counted as correct if they matched any combination of correct alleles. However, if a tool returned multiple predictions, only the top prediction was counted.

Some of the samples in this dataset were also used in the development of the HLA typing tools or at least included in the proof of concept study in the original articles introducing the tools. Out of the 829 individuals, 31 were included in the paper introducing Kourami, 28 for HLA*LA and 95 in the paper introducing Optitype. The tools were, however, not specifically designed to type these samples. Instead, the samples were included in the papers as part of smaller benchmarking studies that demonstrated the performance of each tool. We, therefore, chose to keep these samples in this benchmarking study.

### 2.3 Performance evaluation

The five HLA typing tools were evaluated on several different metrics with the most important being the typing accuracy, which is the number of correct predictions out of the total amount of HLA alleles. The typing accuracy was found for the resolutions: 1-field, pseudo-sequence, P group and 2-field (see **Figure 1**). Some tools did not return a prediction for all alleles, and the individual tools’ call rate (number of predictions a tool returned as a fraction of the total number of alleles) was therefore also noted. The time (CPU and real), as well as the memory use of each HLA typing tool, was registered and the full pipeline for running the tools is illustrated in **Supplementary Figure S1**.

### 2.4 Conversion between typing resolutions

The reference HLA alleles in the 1000G dataset are in 2-field resolution which cannot be unambiguously converted to G group resolution. This is because 2-field resolution separates alleles based on differences in the full amino acid sequence while G group resolution separates alleles based on genomic differences in the ARD coding exons. 2-field typing is, however, widely used in benchmarking studies and so to make the best estimate of Kourami’s and HLA*LA’s 2-field accuracy, their G group predictions were converted to 2-field resolution by trimming the third field. We found this approach the fairest, as it only allows each tool one guess per allele. The approach is, however, still not perfect, as is discussed in section 4.1.

The pseudosequence resolution, which for HLA-DRB and -DRA is shown in **Figure 1**, was presented by (45) and we use the same approach to constructing the pseudo-sequences as was described in the original article. That is, a pseudo-sequence consists of the 34 amino acids, which are within 4 Å of a peptide bound to the HLA molecule. Alleles which share these 34 amino acids belong to the same pseudo-sequence group.

### 2.5 Downsampling

The 230 samples in the 1000G dataset with a DOC of at least 100X were included in the downsampling study. Downsampling was performed by first finding the full sequencing depth of the CRAM files used in the 1000G dataset. This was done using *mosdepth* (version 0.2.6) (46). Hereafter, alignment files containing potential HLA reads were downsampled to 1X, 2X, 5X, 10X, 20X, 50X, 75X and 100X using *samtools view -s* (47). The resulting files were then HLA-typed in the same way, as in the main study (see **Supplementary Figure S1**).

### 2.6 Optitype’s performance on simulated ancient DNA

The 50 WES samples with the highest DOC from the 1000G dataset were used to simulate an aDNA dataset. Samples were downsampled as described above. Gargammel (v. 1.1.2) (48) was used to trim reads to specific read lengths and to add simulated chemical damage. The added chemical damage was done in a way to simulate the chemical damage in the samples from a 2021 study of medieval plague victims (49). Specifically, the added damage was 20 base of C to T substitutions after the 5’ end, the substitution rate for the base immediately after the 5’ end was 7% and fell below 1% 3 bases after the 5’ end. There were also 20 bases before the 3’ end of G to A substitutions, the base right before the 3’ end has a substitution rate of 6% and then fell below 1% 3 bases before the 3’ end. Reads were downsampled to a DOC of 1X, 2X, 5X, 10X, 20X and 50X and read lengths were varied over 10, 13, 15, 20, 25, 30, 35, 45, 55 and 65 base pairs. The WES samples did not have enough reads to achieve a DOC of 50X for the lower read lengths, so some read length/DOC combinations could not be performed.

## 3 Results

This section will focus primarily on the clinically relevant P group resolution and the typing results as well as the HLA types noted in the 1000G dataset are available (in P group resolution) in **Supplementary Table S2**. All results presented in this section are, however, available both as raw typing output from the HLA typing tools and converted to four typing resolutions on this project’s GitHub^8^.

### 3.1 Overall performance of the tools

**Figure 2:**
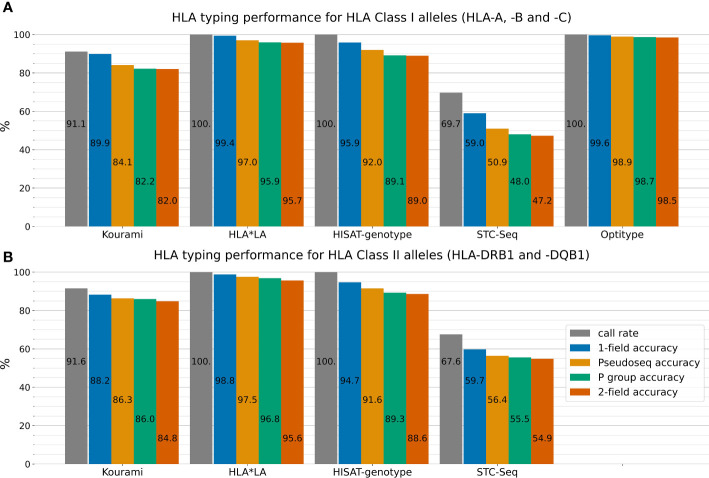
The five HLA typing tools’ typing accuracy (number of correctly called alleles out of the total amount of alleles) and call rate (number of called alleles out of all alleles) in 1-field, pseudo-sequence, P group and 2-field resolution for the three HLA Class I genes HLA-A, -B and -C **(A)** and the two HLA Class II genes HLA-DRB1 and -DQB1 **(B)**. Optitype does not offer Class II typing and is therefore not listed in **(B)**. Note, that the typing accuracy is not relative to the call rate, but to the total amount of alleles. The call rate is displayed alongside the typing accuracy to depict the difference between an allele not being typed by a tool and a tool mistyping an allele. For example, Kourami offers correct 1-field typing for 89.9% of Class I alleles, but only a smaller part of the missing 10.1% is due to Kourami returning a wrong prediction. Instead, the majority of miscalls can be attributed to Kourami not returning any prediction at all. **Supplementary Figure S2** shows these results stratified on the five individual HLA genes.

**Figure 2** outlines the performance of the five HLA typing tools on the full 1000G dataset across four different typing resolutions. HLA*LA, Optitype and HISAT-genotype all have a call rate of 100% across the genes that they offer predictions for. Kourami fails to return a prediction for almost 9% of alleles and STC-Seq for more than 30%. In Kourami’s case, a failure to return a prediction often happens when there is not enough data to sufficiently cover important regions leading to Kourami’s graph structure being disconnected (21). Optitype is the best performing of the tools for Class I genes and has a typing accuracy close to 100% across all typing resolutions. These results match the results of previous studies evaluating the performance of Optitype (25, 26). HLA*LA is the best performing tool for the two HLA Class II genes as well as across all five HLA genes.

HLA*LA performs almost as well as Optitype at 1-field resolution, while the difference in performance between the two tools is larger at higher typing resolutions. In 2-field resolution, HLA*LA mistypes 4.3% of Class I alleles while Optitype only mistypes 1.5%.

For both HLA Class I and Class II genes, the typing resolution does not change which tools perform the best. Across all typing resolutions and Class I genes, Optitype has the highest typing accuracy, HLA*LA has the second-highest followed by HISAT-genotype, Kourami and STC-Seq in that order. The order remains the same across the Class II genes for the tools that offer Class II typing. Using P group resolution instead of 2-field does however make some difference to the typing accuracy. HLA*LA miscalls 141 out of 3316 Class II alleles in 2-field resolution, but 36 of the 141 are correct calls in P group resolution and 59 of the 141 are correct calls in pseudo-sequence resolution.

**Figure 3:**
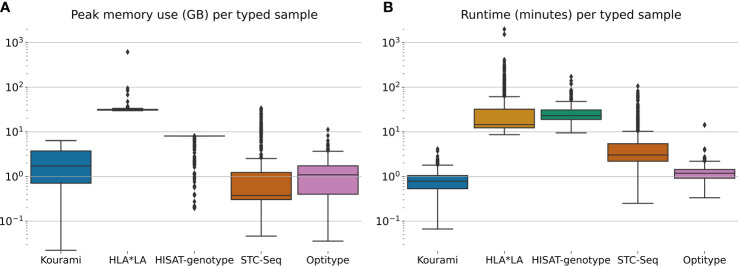
**(A)** The peak memory usage and **(B)** real-time usage of HLA typing for each sample in the full 1000G dataset. Note that this is only for the tool-specific HLA typing step and does therefore not include any of the previous steps such as the extraction of HLA reads.

**Figure 3** shows the distribution of the peak memory usage and real-time usage across the 829 samples in the 1000G dataset for each of the five tools included in this study. Generally, STC-seq, Optitype and Kourami use the least memory per sample, with median usages of 0.38 GB, 1.1 GB and 1.7 GB respectively. For a few of the samples, STC-Seq and Optitype use more than 8 GB of memory, while the most memory-demanding sample takes 6.4 GB for Kourami. HISAT-genotype uses 8 GB of memory for almost all samples, indicating that this is a built-in restriction. Allowing HISAT-genotype to use more than 8 GB of memory could perhaps reduce the runtime of the tool. HLA*LA uses by far the most memory with a median of 31 GB per sample and the most memory-demanding sample (NA18504) requiring over 600 GB of memory. The high memory usage is due to HLA*LA’s expensive alignment step that uses dynamic programming (21) and has been noted by the HLA*LA developers^9^.

HLA*LA and HISAT-genotype spend the most time per typed sample. HISAT-genotype’s median time (23 minutes) is higher than HLA*LA’s median time usage (15 minutes) but HLA*LA spends more than a day for a few samples, whereas HISAT-genotype at most spends 172 minutes. STC-seq spends more than an hour for some samples, but types most samples in under 10 minutes. Kourami and Optitype type most samples in less than 2 minutes. The CPU time usage of the tools can be found in **Supplementary Figure S3**.

Kourami, Optitype, HISAT-genotype and STC-seq can all run on a system with less than 16 GB of memory and run most samples in less than an hour with Optitype and Kourami generally requiring far less time. HLA*LA requires much more memory than the other tools and spends more than an hour for 129 samples and more than 24 hours for 2 samples. These high resource requirements should be kept in mind when choosing this tool.

### 3.2 Systematic errors in the typing results and in the 1000G dataset

Some HLA alleles are very common and others are so rare that they have only been detected in one individual. Allele frequencies further differ between ethnic groups and some alleles are common in one population group but rare in another (7). To assess whether the 1000G dataset used in this study is a fair representation of a global population, we compared the allele frequencies in the 1000G dataset with that of the US donor registry dataset presented by (50), which contains HLA typing data from 2.9 million individuals distributed across 21 population subgroups.

**Supplementary Figure S4** shows a comparison of the allele frequency distribution of the 1000G dataset and that of the US donor registry dataset by (50). There are some differences between the allele frequencies in the two datasets; DRB1*15:02, for example, has an allele frequency of almost 2.5% in (50) but less than 0.5% in the 1000G dataset. On an overall scale, however, the allele frequencies in the 1000G dataset are similar to the allele frequencies in (50) and a paired t-test comparing the two allele frequency distributions did not show that the two datasets differed significantly (p=0.72). It has been noted that allele frequency data for rare alleles is generally less robust than that of common alleles and that, when working with reference population data, focusing on the more common alleles may be a better approach (7). The 1000G and US donor registry frequency distributions are more alike for common alleles than rare alleles, and this approach would therefore not change the prior conclusion.

To get an overview of alleles, for which the individual HLA typing tools had a particularly low typing accuracy, we calculated the typing accuracy (P group resolution) for each tool for each unique allele in the 1000G dataset. This is shown in **Supplementary Table S2**, which for each entry also notes the allele frequency in the 1000G dataset and the allele frequency for individual population groups in the US donor registry. A notable allele is A*11:01, which Optitype types correctly in 113 out of 113 cases but HLA*LA only correctly types in 79 cases. Another is DRB1*03:02, which HLA*LA correctly types in all 33 cases, Kourami correctly types in 32 cases, but HISAT-genotype only correctly types in 6 cases.

**Supplementary Figure S4** shows that the 1000G dataset contains both rare and common alleles and it could be hypothesised that the tools would perform better on frequent alleles than on less frequent alleles. This is, however, not generally the case as shown in **Supplementary Figure S5**. This figure shows that for Kourami, HLA*LA and HISAT-genotype, the mistyped alleles have a higher median allele frequency (as defined by the US donor registry) than the median allele frequency of the correctly typed alleles. For Optitype, the median frequencies for correctly typed and mistyped alleles are almost identical. Another way of looking at allele frequency is to separate the alleles into rare and common alleles and compare the performance of the tools on these two groups. We defined an allele as “rare” if it had an overall frequency of less than 1/2000 across all individuals in the US donor registry dataset. Alleles with a higher frequency than 1/2000 were defined as “common” alleles. This definition is similar to the one made in (50). **Supplementary Table S3** lists the overall typing accuracy for each tool for rare and common alleles, respectively. Kourami and HLA*LA have a higher typing accuracy for the rare alleles (88.6% and 100%) than for the common alleles (83.7% and 96.3%), while HISAT-genotype and Optitype have a much higher typing accuracy for the common alleles (89.1% and 98.8%) than for the rare alleles (61.4% and 92.1%). Note that Optitype’s typing accuracy here is only calculated for HLA Class I genes.

We expanded upon the analysis of rare alleles by looking into whether the HLA typing tools performed better for alleles common (>=0.0005%) to Caucasians compared to rare alleles (<0.0005%) in Caucasians, but common in one of the other four ethnic groups (African American, Asian/Pacific Islander, Hispanic or Native American). If this was the case, it could potentially be a result of the genomic reference dataset being biased towards Caucasian data, as has been discussed in recent studies (51). **Supplementary Figure S6** shows that Kourami, HLA-LA and HISAT-genotype actually performed worse on the alleles common to Caucasians, whereas there was no notable difference in typing accuracy between the two groups for Optitype.

We also investigated whether there were any general differences in typing accuracy between the four population groups represented in the 1000G dataset (European, African, Ad Mixed American, and East Asian). **Supplementary Table S4** shows an overview of the typing accuracy for each tool across the five HLA loci and the four population groups. For HLA Class I loci and HLA-DRB1 there is no clear indication that the tools favor or disfavor a specific population group. However, Kourami, HLA*LA and HISAT-genotype are all remarkably inadequate at typing HLA-DQB1 from East Asians compared to their performance for the other three population groups. HLA-LA has an error rate of more than 11% for East Asians HLA-DQB1, and an average error rate of less than 3% for the other three population groups. Kourami only has a typing accuracy below 40% for East Asian HLA-DQB1, but an average of above 80% for the three other population groups. Note here, that a large part of Kourami’s miscalls for HLA-DQB1 can be attributed to Kourami not converging to a solution and not returning a result (see **Supplementary Figure S2**). **Supplementary Figure S7** shows a comparison of the DOC of the four population groups across all sequenced exomes as well as specifically the ARD coding region (exon 2) of HLA-DQB1. The East Asian samples have a higher DOC across all sequenced exomes than the samples from the other population groups but a number of the East Asian samples have an especially low DOC for the ARD coding region (exon 2) of at least one of their HLA-DQB1 alleles.

As shown in the 2018 study, the dataset presented by (42) likely contains some errors and, even with the adjustments to the dataset described in section 2.2, the 1000G dataset may still harbor some errors. **Supplementary Table S5** lists the typing results for 54 loci in the 1000G dataset, where a majority of the well-performing HLA typing tools (Kourami, HLA*LA, HISAT-genotype and Optitype) mistyped at least one of the alleles. For 5 of these samples (NA18507, NA19130, NA19131, HG00610 and HG00625), a majority of the tools agreed on a typing, which differed from the one noted in the 1000G dataset. The fact that a majority of the tools mistype these samples could be due to difficulties in distinguishing between two very similar alleles, or due to poor sequencing quality, poor primer design or a low DOC specifically for HLA-DQB1, but it may also be due to errors in the 1000G dataset. These samples could be investigated in future studies and perhaps HLA typed using PCR-based methods to improve upon the 1000 genomes HLA dataset.

The 1000 genomes phase 3 dataset contains more WES samples with PCR-validated HLA types, than the samples included in the 1000G dataset used in this study. Due to computational limitations we, however, chose to focus on a subset of individuals. To ensure, that this choice did not impact our results, we verified that that the tools showed similar performances for a random selection of half (414) of the 829 chosen samples (**Supplementary Figure S8**) compared to that of the full 1000G dataset (**Figure 2**).

### 3.3 Downsampling analysis

Previous studies have analysed how the typing accuracy of various HLA typing tools depends on the DOC of the sequencing data (21, 26, 40). Here, we present a more detailed study of how the typing accuracy for each of the tools included in this study depends on the DOC of the sequencing data.

**Figure 4:**
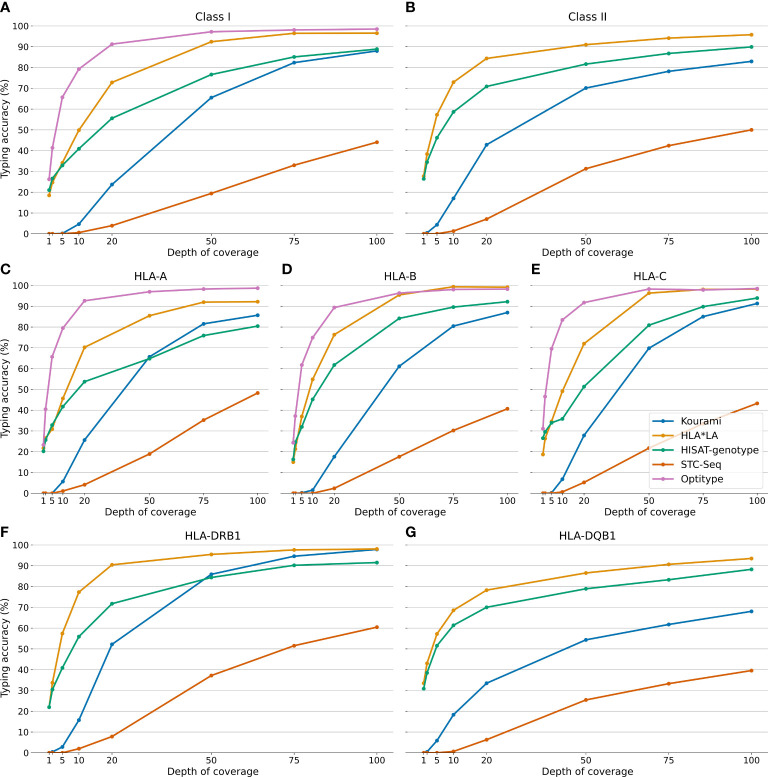
The typing accuracy of Kourami, HLA*LA, Optitype, HISAT-genotype and STC-Seq in P group resolution for 230 WES samples across DOCs ranging from 1X to 100X. The top two panels **(A, B)** show the overall typing accuracy for HLA Class I and II, while figures **(C–G)** show the performance for each individual HLA gene.

The typing accuracies presented in this section are, unless specified, all in P group resolution and therefore match the results shown in **Figure 4**. This figure shows that the typing accuracies of Kourami, HLA*LA, Optitype, HISAT-genotype and STC-Seq depend highly on the DOC of the samples when the DOC varies between 1X and 100X. A higher DOC correlates with a higher typing accuracy but this correlation is not linear and differs between the HLA typing tools. Some HLA typing tools maintain a high typing accuracy when the DOC decreases, while others require a high DOC for accurate typing. Optitype performs the best on samples with low DOC and its typing accuracy only drops below 90% when the DOC is below 20X. HLA*LA, which performs almost as well as Optitype at 100X, has a typing accuracy of 72.7% at 20X for the Class I genes. STC-Seq and Kourami are both reported to have a typing accuracy very close to 100% when typing from each tool’s preferred sequencing data (HLA enriched data for STC-Seq and high DOC WGS data for Kourami) (21, 22) but, as is shown in this study, the tools do not perform well on WES data with a low DOC.

**Figures 4C–G** show that the HLA typing accuracy not only varies between tools and with DOC, but also between HLA genes. HLA typing tools in this study achieve a higher typing accuracy for HLA-DRB1 than they do for HLA-DQB1. At 100X, HLA*LA has a higher typing accuracy than Optitype for HLA-B, and HISAT-genotype is much better at typing HLA-B and -C than HLA-A. The difference in performance between HLA-DRB1 and HLA-DQB1 is especially prevalent for Kourami. At a DOC of 100X, Kourami has a call rate of 100% and a typing accuracy of 97.8% for HLA-DRB1, while for DQB1 the tool only has a call rate of 78.2% and a typing accuracy of 68.0%.

The gain in typing accuracy from increasing the DOC is, as expected, generally larger when the DOC is low, and there are diminishing returns from an increase in DOC when the DOC is already high. This trend, however, depends on the HLA typing tool and the HLA gene. For the HLA Class I genes, Optitype and HLA*LA do not benefit much from an increase in DOC from 75X to 100X, while the remaining three tools might even benefit from increasing the DOC beyond 100X. For HLA-DRB1, HLA*LA and HISAT-genotype’s perform almost equally well at 75X and at 100X but for HLA-DQB1, the tools perform notably better at 100X than 75X. HISAT-genotypes typing accuracy even increases more from 75X to 100X than it does from 50X to 75X.

**Supplementary Figure S9** shows how the memory usage, runtime and CPU time usage depend on the DOC of the samples. Generally, the DOC has little impact on the memory usage whereas it greatly affects both the runtime and CPU time usage of HLA*LA and HISAT-genotype, indicating that these tools would require significantly more time to type a sample at 200X than what is shown in **Figure 3**.

The findings from the downsampling dataset generally agree with those from the full dataset, but there are some notable differences. For the full dataset, Optitype has the highest typing accuracy for all three Class I genes (see **Supplementary Figure S2**), but for the downsampling dataset, HLA*LA outperforms Optitype for HLA-B and is as good as Optitype for HLA-C when the DOC is above 75X. Kourami has a higher typing accuracy than HISAT-genotype for HLA-A on the full dataset, but the downsampling dataset shows that HISAT-genotype has a higher typing accuracy when the DOC is below 50X. Kourami also shows a large improvement with an increase in the DOC for HLA-DRB1 and at 100X it performs almost as well as HLA*LA.

### 3.4 The impact of short fragments and DNA damage

**Figure 5:**
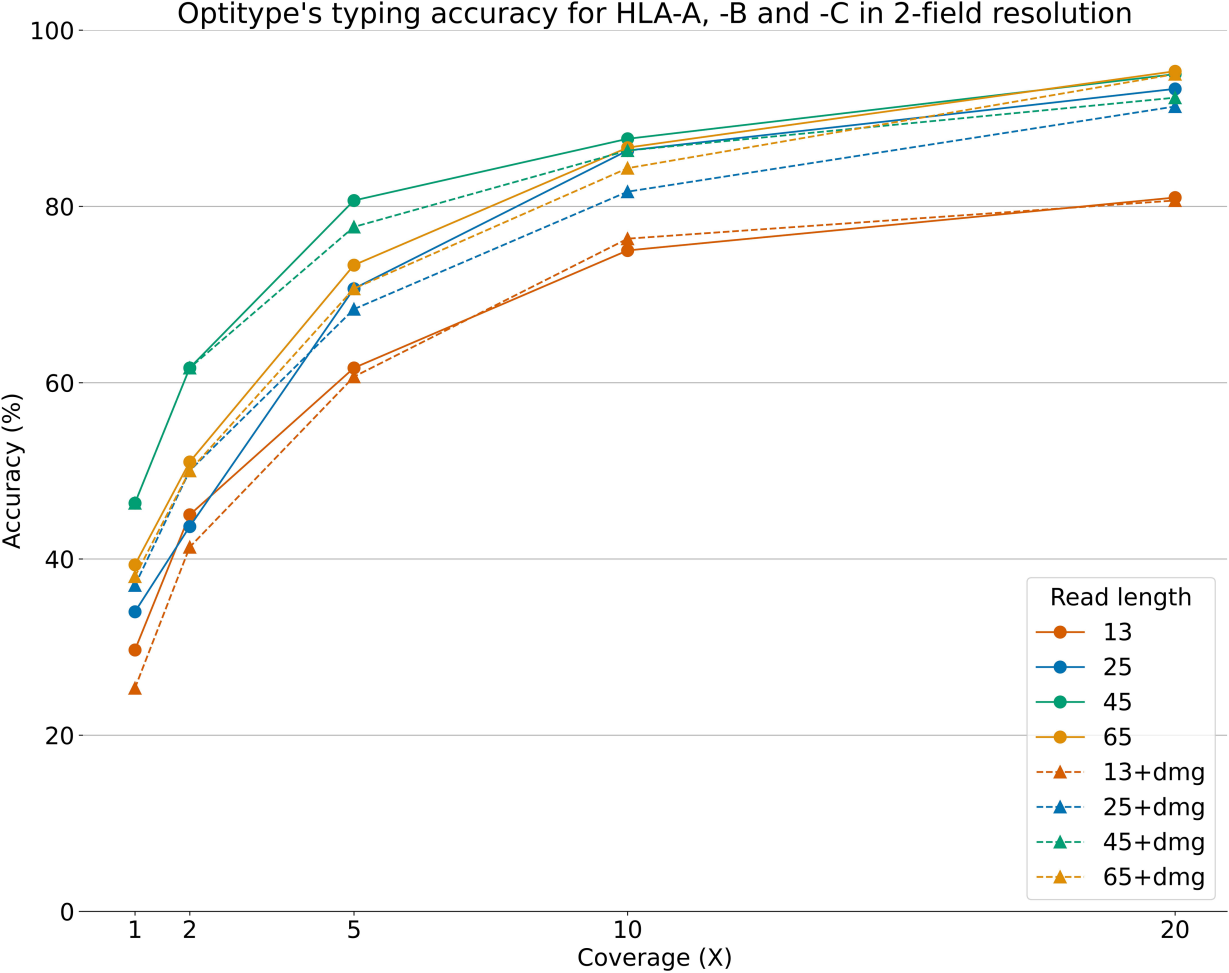
Optitype’s typing accuracy for HLA-A, -B and -C across 50 samples and across varying read lengths, DOCs and with/without artificially added chemical damage. The typing accuracy depends primarily on the DOC. Note that the downsampling, read cutting and addition of DNA damage was done after an extraction of HLA reads. This figure therefore represents a best case scenario, where assigning reads to the HLA region is as accurate for short reads as it is for the full length reads.

**Figure 5** shows Optitype’s expected performance across varying DOCs and read lengths, with/without damage added to the reads. The figure only shows a selection of the combinations between DOC/read length and added DNA damage. The full results are available on this project’s GitHub. We found that Optitype was unable to return any results when the read length was 10, regardless of the DOC. As also shown in **Figure 4**, Optitype’s typing accuracy depends largely on the DOC when it varies between 1X and 20X. At lower DOCs, the read length seems to have some influence on typing accuracy, but this effect vanishes when the DOC increases. Between 1X and 10X, Optitype performs the best when the read length is at 45, and both lower and higher read lengths result in a drop in typing accuracy. There is still a slight performance gain by increasing the DOC from 20X to 50X, as shown in **Supplementary Figure S10**, but at this stage, an increase in read length does (as long as it is above a minimum of around 25) not result in a higher typing accuracy.

The addition of simulated DNA damage did not notably impact the typing accuracy at any DOC or read length. The typing accuracy in **Figure 5** and **Supplementary Figure S10** is shown in 2-field resolution, as this resolution was used in (32).

Besides providing the HLA typing for a sample, Optitype also returns the number of sequencing reads, which its optimal solution explains. **Supplementary Figure S11** shows how the typing accuracy correlates with the median number of explained reads for a selection of the combinations of read length and DOC described in section 2.6. Unsurprisingly, the median number of explained reads correlates well with the typing accuracy in a similar fashion to how the DOC correlates with the typing accuracy. Additionally, for a set number of explained reads, a higher read length gives a higher typing accuracy. This is also expected since 100 reads with read length 45 contains more information than 100 reads with read length 25.

The figure additionally shows how a higher number of explained reads can make up for a lower read length. At a DOC of 10X, Optitype’s median number of explained reads was 465 at a read length of 25, 257 for a read of 45, and 139 for a read length of 65. The typing accuracy was between 86 and 88% for all these three cases. However, if the read length is too low the typing accuracy is affected. At a DOC of 10X and a read length of 13, Optitype’s median number of explained reads was 1081, but the typing accuracy for this group was only 75%.

## 4 Discussion

This study investigated the performance of five NGS-based HLA typing tools: Kourami, HLA*LA, HISAT-genotype, Optitype and STC-Seq, for the five HLA genes: HLA-A, -B, -C, -DRB1 and DQB1. The tools were evaluated on 829 WES samples from the 1000 genomes dataset as well as on a downsampled subset of these to evaluate the impact of the DOC on typing accuracy. The typing accuracy was evaluated at four different typing resolutions: 1-field, 2-field, P group and pseudo-sequence, with the two latter not explored in previous studies of the performance of HLA typing algorithms.

HLA*LA was found to have the highest overall typing accuracy (96.3% in P group resolution for the full dataset) and the highest typing resolution for the two HLA Class II genes (96.8% in P group resolution), while Optitype was found to have the highest typing accuracy for the three HLA Class I genes (98.7% in P group resolution). The tools varied greatly in computational resource consumption with HLA*LA requiring 30 GB of memory and often more than an hour per typed sample, whereas Optitype only required 1 GB of memory and rarely more than a couple of minutes.

Evaluating the HLA typing on samples across varying DOCs showed that the typing accuracy of all the tools depended greatly on the DOC, although this dependency differed between both the HLA typing tools and across the five HLA genes. A DOC of 50X was satisfactory for Class I typing using Optitype, while accurate typing of HLA-DQB1 required at least a DOC 100X even for the best performing tool, HLA*LA.

### 4.1 Issues related to allele conversion between G group and 2-field resolution

In this study, we converted predictions from HLA*LA and Kourami in G group resolution to 2-field resolution by simply removing the third field. However, converting predictions from G group to 2-field and vice versa is ambiguous, as G group resolution focuses on the DNA sequence of the ARD coding exons, while 2-field resolution focuses on the amino acid sequence of the full HLA molecule.

There are examples of HLA molecules (specific alleles in 2-field resolution) which are part of two G groups that differ on the second field. An example is HLA-C*02:02, as this could both be C*02:02:02:01 (which belongs to the C*02:02:02G), C*02:02:01 (which is not part of a G group) or even C*02:02:37 (which is part of the C02:10:01G group that differs from C*02:02 at the second field).

Conversely, there are examples of G groups containing alleles which differ in 2-field resolution. The G group HLA-A*01:01:01G contains A*01:01, but also A*01:32 and 78 other alleles that differ in 2-field resolution. This ambiguity poses a problem when using typing methods such as HLA*LA and Kourami, which return the results in G group resolution, but also when evaluating their performance on the 1000G dataset in 2-field resolution. For example, if the correct allele was HLA-A*01:32, the correct prediction in G group resolution would be A*01:01:01G. Should A*01:01:01G be counted as correct in 2-field resolution even though the prediction, besides A*01:32, could refer to more than 70 individual alleles in 2-field resolution? One approach to this ambiguity is to convert both the reference alleles and all predictions to G group (e.g. HLA-A*01:01:01G) and then trim to 2-field resolution (HLA-A*01:01), but this cannot be done unambiguously for alleles such as C*02:02, as described previously. A similar approach which partly solves the ambiguity issue is to convert predictions to P group resolution. 2-field and G group resolution can, except for null alleles, both unambiguously be converted to P group resolution.

The approach used in this study to convert G group predictions to 2-field resolution allows for a somewhat fair tool comparison in 2-field resolution, despite the aforementioned ambiguity.

Another factor impacting the accuracy of the results in 2-field resolution is that the experimental method for discovering the gold standard HLA types of the 1000G dataset used by (42) only focuses on the ARD coding exons. Therefore, the experimental method cannot be used to distinguish between alleles which differ in 2-field resolution because of differences in non-ARD coding exons. If e.g., an allele is typed as C*07:01 in the 1000G dataset and a tool predicts the allele to be C*07:18, this will count as a wrong prediction. C*07:01 and C*07:18, however, share identical ARD coding exons and the typing method used by (42) can therefore not be used to distinguish between them, which means that the correct 2-field resolution actually could be C*07:18. For some samples, this is partly mitigated by noting an ambiguous typing. The example mentioned in section 2.2 is NA12287 with the HLA-B typing: 15:01/15:03 and 15:01/15:26/15:12/15:19. As with the problem with G group/2-field conversion, this problem can also be solved by converting both the 1000G dataset and the predictions to P group resolution.

### 4.2 Sufficient depth of coverage is crucial for accurate HLA typing

A 2013 study (52) stated that the DOC of many of the samples in the 1000 genomes dataset was too low for HLA typing. Another article from 2018 disputed this statement and stated that they found no correlation between typing accuracy and DOC, although noting that a minimal DOC was required (26). The results outlined in **Figures 2**, **4** clearly show that even the samples with the lowest DOC in the dataset (between 35X and 40X) can be typed accurately if a tool suited for low DOC sequencing data is used. This is in contrast to the results found by (52). The results also show that the typing accuracy depends on the DOC of the sample, but to a lower degree when the DOC is high. Furthermore, the required minimal DOC for accurate HLA typing depends on both the HLA typing tool and the HLA gene.

Optitype and HLA*LA’s overall performances do not notably improve when the DOC is increased from 75X to 100X. However, the three other tools do improve, and may even see an improvement if the DOC is increased beyond 100X. Achieving a typing accuracy above 90% for HLA-DQB1 from WES data also requires DOC of at least 100X, even for the best performing tool, HLA*LA. These results align roughly with minimal DOC recommendations for clinical WES of 120X (53) and WES-based HLA typing of 100X (40). The results from this study are however more detailed and indicate that for some genes, e.g. HLA-DQB1, or tools, e.g. Kourami, it is even beneficial with DOCs above 100X.

The DOC of the samples in the 1000G dataset varies between 37X and 456X, with many samples having a lower DOC than what is required for optimal typing. The tools’ performances on this dataset might therefore not be an accurate estimate of the performances on clinical WES with a DOC above 100X. A better estimate of this could be the downsampling results, specifically the performance of the samples at 100X. This is a redeeming factor for e.g. Kourami, which had a mediocre performance on the full dataset, but where the results of the downsampling study show that the lower DOC of the full dataset likely impaired Kourami’s performance much more than it did Optitype’s.

HLA*LA is the best performing tool on average, across the five HLA alleles but the tool does require an extensive amount of memory and is relatively slow. Mistypings/mismatches at HLA-DQB1 are less critical than mistypings at HLA-A, -B, -C or -DQB1 (54), hence Optitype is a lighter and faster alternative to HLA*LA for Class I typing and, assuming a DOC of at least 100X, Kourami as an alternative for Class II typing. For the 230 samples in the downsampling study at a DOC of 100X, Optitype had a P group typing accuracy of 98.3% across the three Class I genes (HLA*LA had 96.4%) and Kourami had a P group typing accuracy of 97.8% for HLA-DRB1 (HLA*LA had 98.0%).

### 4.3 Systematic errors with DQB1 typing for East Asians in the 1000 genomes dataset

As discussed in section 3.2, the HLA typing tools are suboptimal at typing HLA-DQB1 for the East Asian samples. The outline of the DOC of HLA-DQB1 presented in **Supplementary Figure S7** indicates that at least part of the reason for the low typing accuracy for HLA-DQB1 for the East Asian samples could be the lower DOC for HLA-DQB1 for these samples. This could be a result of poor primer design in the exome sequencing. The low typing accuracy could, however, also be due to poor probe design in the sequencing to obtain the reference genotype in the 1000G dataset, resulting in errors in reference dataset used in this study.

Regardless, if the reason for the poor performance for HLA-DQB1 is specifically due to some errors in the 1000G dataset and not a fault of the HLA typing tools then the findings of this study regarding typing of HLA-DQB1 could be re-evaluated. Future studies of HLA-DQB1 relying on the 1000 genomes database would likely benefit from omitting the East Asian samples until the cause of the problem has been identified.

### 4.4 Optiype’s performance on ancient DNA samples with varying read length and sequencing coverage

In **Figure 5**, we present the first benchmarking of HLA typing tools on aDNA. The figure shows that the DOC is the most determining factor for high typing accuracy but, at low DOC, both a too high or a too low read length can impair typing accuracy. It is expected that a low read length negatively affects allele typing - especially for a highly polymorphic region, such as the HLA region. However, it is surprising that the typing accuracy for the samples with a read length of 65 is lower than when the read length is 45. One reason for this might be that Optitype’s typing algorithm creates a binary hit matrix where the predicted HLA alleles explain the highest *number* of reads. When the read length is increased, and the DOC is kept constant, the number of reads decreases. The sample with reads of length 45, therefore, contains more reads than the one with a read length of 65 at the same DOC as is illustrated in **Supplementary Figure S12**. The difference in performance, which can be attributed to read length, decreases at higher DOC and the typing accuracy is almost the same at a DOC of 10X.

The addition of DNA damage did not impair the performance notably. However, the DNA damage applied in this study corresponded to that of specific samples from the 16th century and older samples or samples from individuals stored in different conditions can have a larger degree of DNA damage, which could have a bigger effect on the accuracy of the HLA typing.

Studies of aDNA often use methods designed for contemporary data for variant calling, which can lead to inaccurate results (55). (32) performed a genomic analysis of individuals who lived around 3200 BCE and part of this was an HLA analysis of 23 individuals where they observed notable shifts in allele frequencies. The study used Optitype for HLA typing in combination with another method but did so without investigating Optitype’s expected performance at the DOC found in their aDNA dataset. The median DOC of the samples, which were HLA typed in the study, ranged from 0.07X to 18.2X with a median of 4.3X. The average read length spanned from 51.8 to 67.6 with a median of 58.2. Our results outlined in **Figure 5** show that most of the samples included in (32) had such low DOC that Optitype likely only returned a correct prediction for little over half of the alleles. The study did not rely solely upon Optitype for HLA typing, but our findings show that Optitype’s typing results are not reliable unless the aDNA samples have sufficiently high read length and sufficiently high DOC.

### 4.5 Summary of findings

This study presents the first analysis of the performance of NGS based HLA typing tools in P group and pseudo-sequence resolution. It further compares these results to the performances of the tools in 2-field and 1-field resolution and offers a discussion on the benefits of P group resolution compared to the commonly used 2-field resolution.

Additionally, the current study offers a detailed outline of how each individual HLA typing tools’ performance is affected by the DOC of the WES sample for each of the five HLA loci HLA-A, -B, -C, -DRB1 and -DQB1. Previous studies have recommended a minimal DOC of 100X-120X for WES samples for clinical use. The findings of this study generally supports the previous recommendations, but also outlines the consequences of using WES data with a lower DOC and provides examples of tools/loci, where sequencing data with a lower DOC could be sufficient and tools/loci where a higher depth of coverage is advisable.

Interestingly, we further found, that all tools were not accurately typing HLA-DQB1 for East Asian samples compared to any other population group. These samples could be investigated in a future study to find the cause of this difference in performance.

Lastly, this study offers the first structured analysis of Optitype’s performance on aDNA across varying DOC and with/without artificially added chemical damage. This tool has been used in previous aDNA studies and will likely also be used in future studies.

## Data availability statement

The datasets presented in this study can be found in online repositories. The names of the repository/repositories and accession number(s) can be found in the article/**Supplementary Material**. The code associated with this project is available at https://github.com/nikolasthuesen/hla-typing-benchmark.

## Author contributions

NT, MK, SG, TT and GR conceived this study. NT gathered the data, installed and ran the tools, performed the subsequent data analysis and wrote the manuscript. MK and GR provided support to the data analysis, GR assisted with the analyses related to ancient DNA. All authors contributed to the article and approved the submitted version.

## Funding

GR gratefully acknowledges funding from the Novo Nordisk Fonden Data Science Investigator grant ref. NNF20OC0062491.

## Acknowledgments

The authors want to thank Dr Christian Garde from Evaxion Biotech for a variety of help throughout the project, Prof. Anthony Purcell from Monash University for his help in finalising the manuscript and the team behind Denmark’s National Life Science Supercomputing Center (Computerome - https://www.computerome.dk/) for providing the computational resources for this project. This study is based on the NT’s Master’s thesis (56), which can be found at https://findit.dtu.dk/en/catalog/60339f5ad9001d01650f4d5d. This paper previously appeared as a preprint at (57).

## Conflict of interest

NT, MK and TT are employed by Evaxion Biotech and have a financial stake in the company.

The remaining authors declare that the research was conducted in the absence of any commercial or financial relationships that could be construed as a potential conflict of interest.

## Publisher’s note

All claims expressed in this article are solely those of the authors and do not necessarily represent those of their affiliated organizations, or those of the publisher, the editors and the reviewers. Any product that may be evaluated in this article, or claim that may be made by its manufacturer, is not guaranteed or endorsed by the publisher.

## Footnotes

1
http://hla.alleles.org/.

2
https://github.com/FRED-2/OptiType.

3
https://github.com/Kingsford-Group/kourami.

4
https://github.com/DiltheyLab/HLA-LA.

5
https://daehwankimlab.github.io/hisat-genotype/manual/.

6
https://bigd.big.ac.cn/biocode/tools/BT007068.

7
http://hla.alleles.org/alleles/deleted.html.

8
https://github.com/nikolasthuesen/hla-typing-benchmark.

9
https://genomeinformatics.github.io/HLA-PRG-LA/.
