# Supplementary material for: Benchmarking freely available HLA typing algorithms across varying genes, coverages and typing resolutions
Source: Front Immunol. 2022 Nov 8;13:987655. doi: 10.3389/fimmu.2022.987655 (PMC9679531; doi:10.3389/fimmu.2022.987655)
Supplement: Supplementary file 7 [file DataSheet_1.pdf]

Supplementary Material

SUPPLEMENTARY DATA

The supplementary data available for this project can be found at https://github.com/nikolasthuesen/hla-typing-benchmark

SUPPLEMENTARY FIGURES

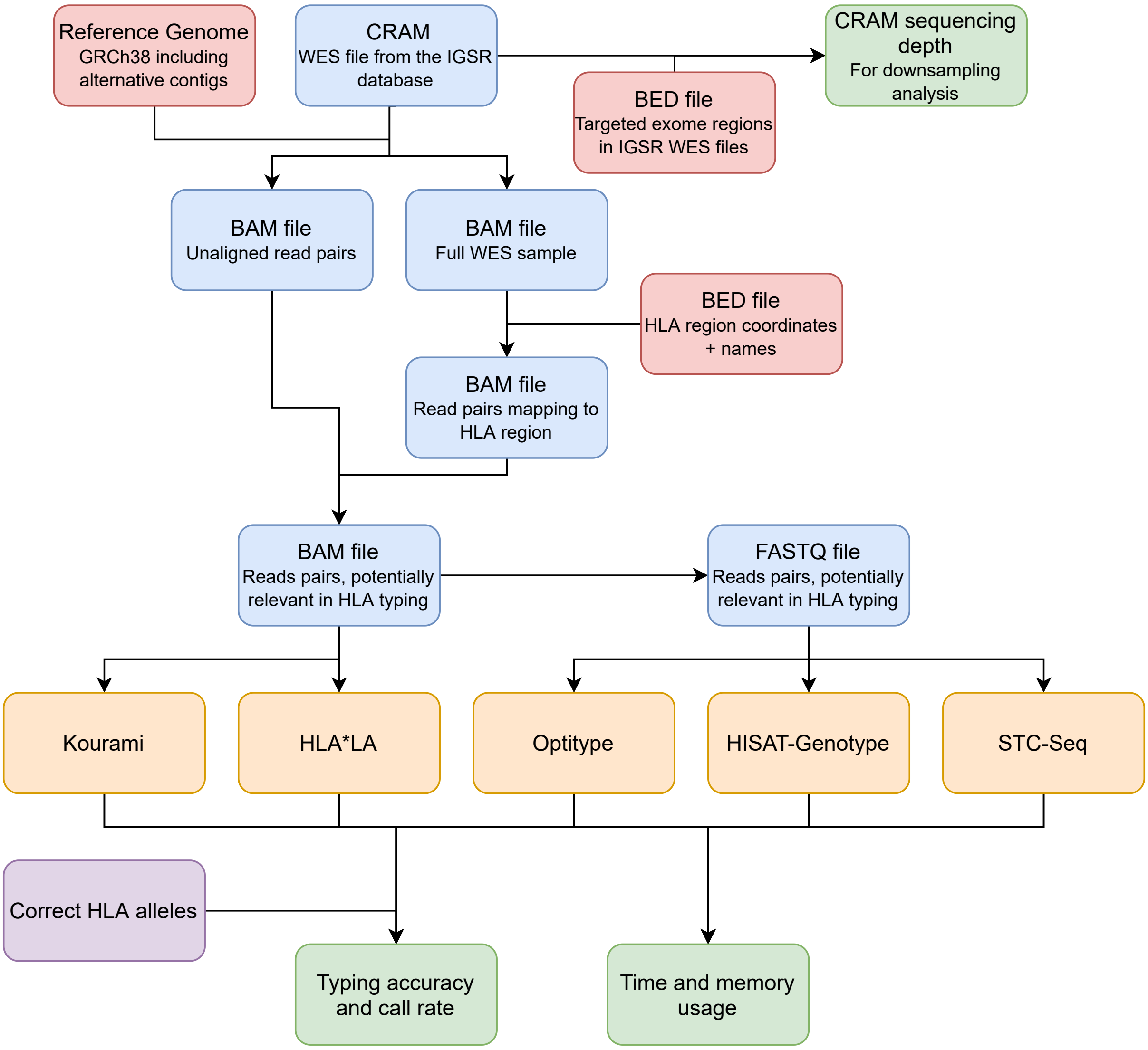

Figure S1. An overview of the benchmarking study. The input CRAM file from the IGSR database (gold standard WES samples) and files deriving from it are shown in blue. These are specific to the individual samples. Reference files which are identical for each sample are shown in red, the HLA typing tools in yellow, the correct HLA alleles for each sample is purple and the output files are all shown in green. For each sample (CRAM file), the sequencing depth is found (used in the downsampling analysis) and the HLA typing tools predict the HLA alleles. Predictions are compared to the correct alleles in the 1000G dataset and the computational resources used are noted for each tool. In the downsampling analysis, the found sequencing depth for each CRAM file is used, but it is not the raw CRAM file that is downsampled. Instead, the reduced BAM file (with read pairs potentially relevant in HLA typing) is downsampled, thereby avoiding the unnecessary extraction of HLA reads in the downsampling analysis. When calculating the use of memory and time, only the step specific to each tool is evaluated. This means that the performance analysis did not include the initial preprocessing measures, the extraction of HLA reads and the conversion of CRAM files to BAM files and further to FASTQ files. Figure created using `https://www.diagrams.net/`

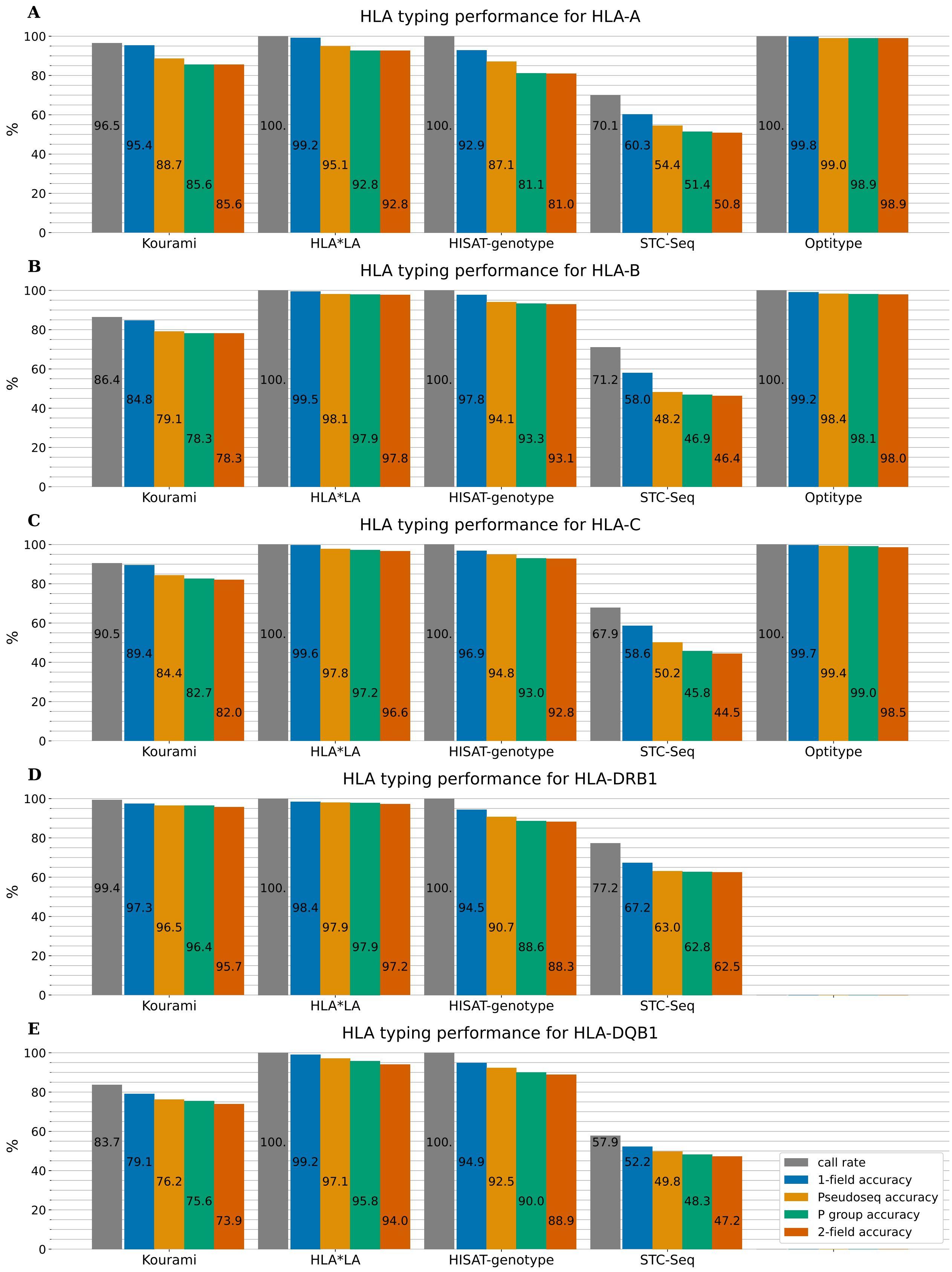

Figure S2. The five HLA typing tools' typing accuracy and call rate in 1-field, pseudo-sequence, P group and 2-field resolution for HLA-A, -B, -C, -DRB1 and -DQB1.

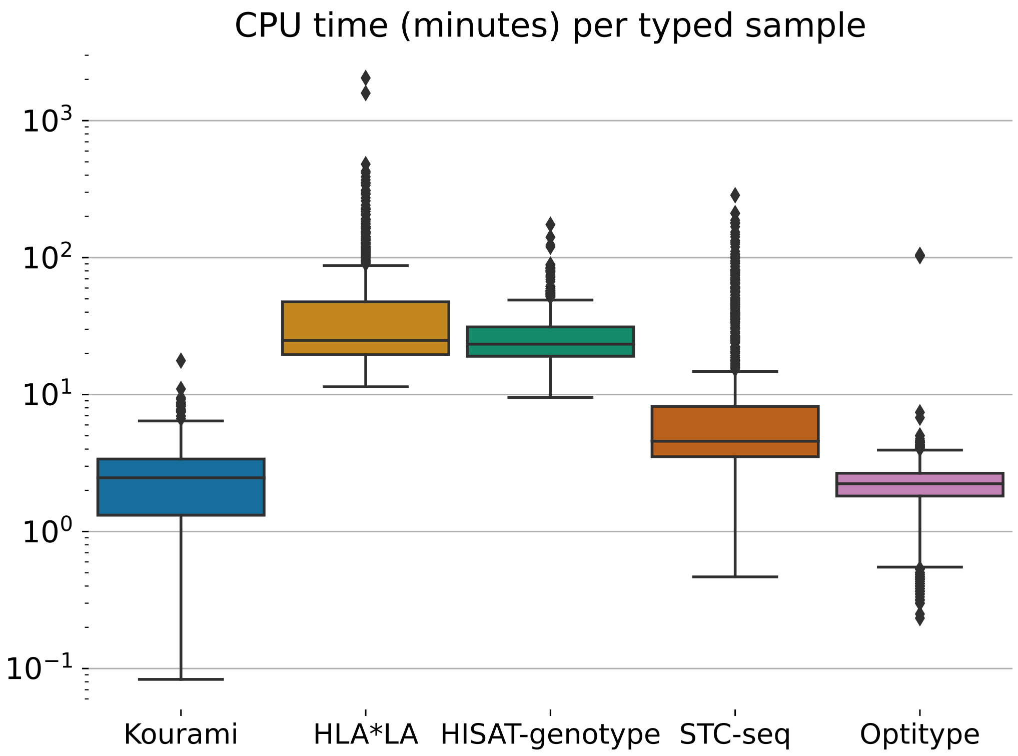

Figure S3. Distribution of CPU time usage for HLA typing for the 829 samples in the 1000G dataset at full coverage. HLA*LA and HISAT-genotype have the highest median CPU time usage (between 20 and 30 CPU minutes), while the rest of the tools have a median less than 10 CPU minutes. For HLA*LA, the CPU time varies a lot between samples. Some samples are typed using a similar amount of resources as is used by Kourami and Optitype, while others require over 100 times more CPU time.

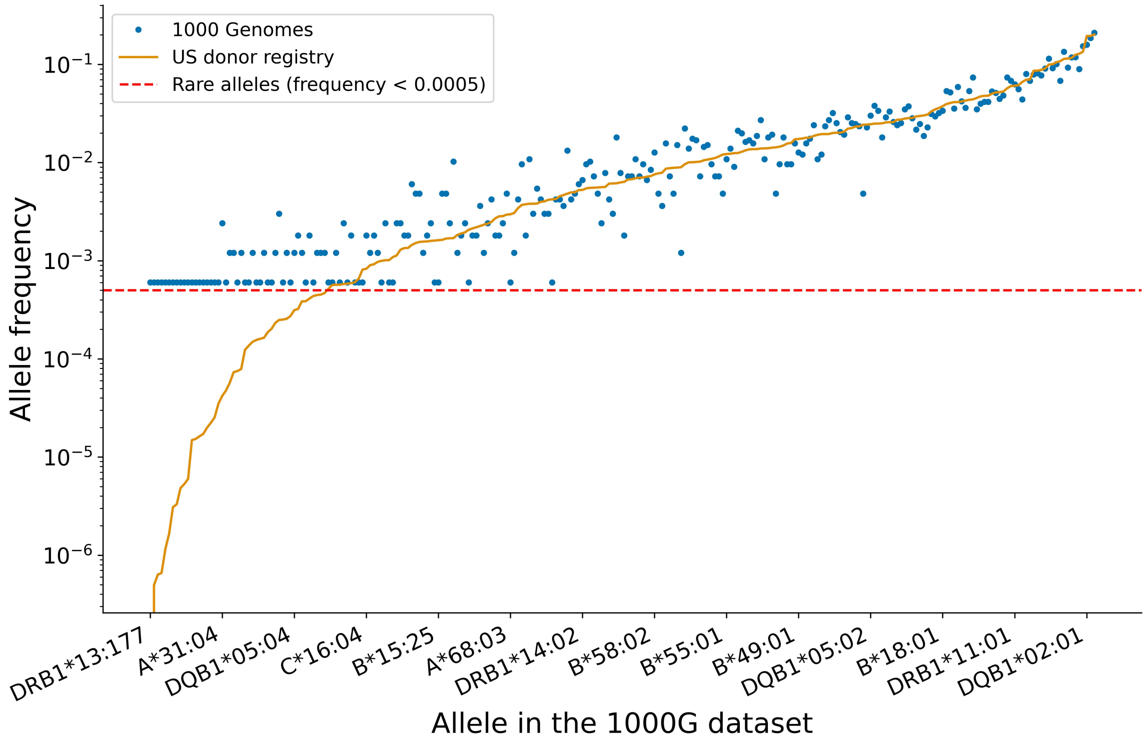

Figure S4. A comparison of the allele frequencies of the alleles 1000G dataset compared to the registered allele frequency for these alleles in the US donor registry dataset. The red line annotates the cutoff point for rare alleles, which is defined as an allele frequency of less than 1/2000. As the 1000G dataset only contains 1658 alleles per locus, no alleles from this dataset fall below this threshold. The two allele frequency distributions generally overlap well - especially for the common alleles.

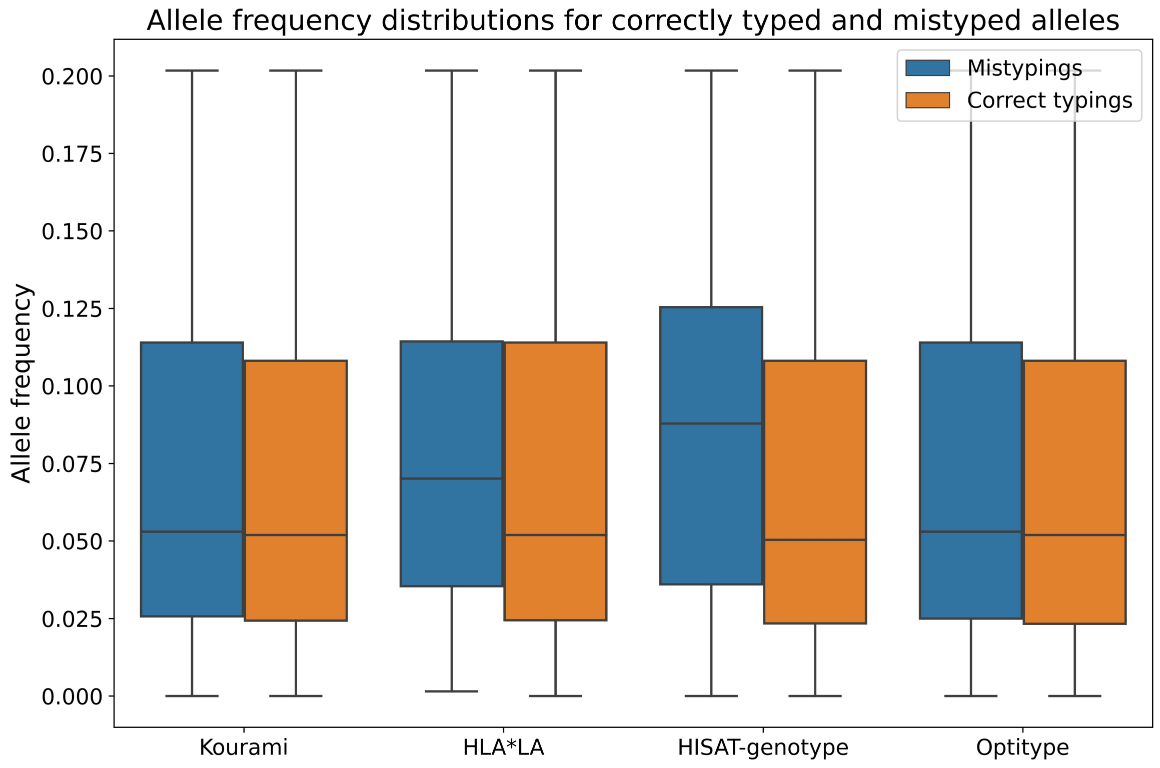

Figure S5. The allele frequency distribution for alleles, which were correctly typed by Kourami, HISAT-genotype, Optitype and HLA*LA. For Kourami, HISAT-genotype and HLA*LA, the median allele frequency for the mistyped alleles is actually higher than that of the correctly typed alleles and for Optitype these two values are almost the same. This hints, that allele frequency and typing accuracy do *not* follow a simple relation, where more frequent alleles have a higher chance of being correctly typed.

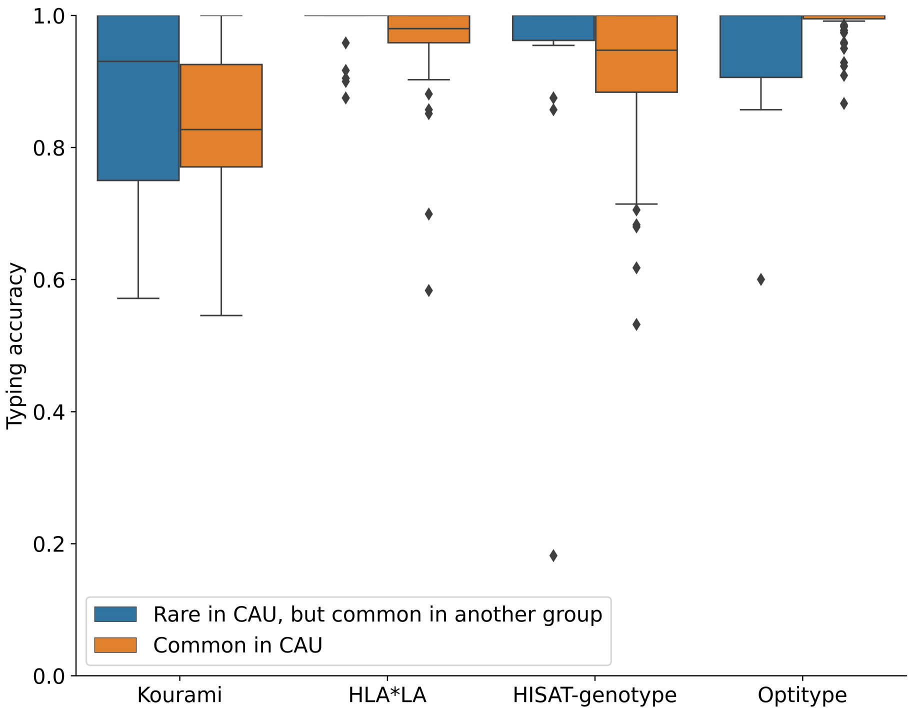

Figure S6. A comparison of the typing accuracy for individual alleles which are either common (>=0.0005%) in a non-Caucasian population but rare (<0.0005%) in Caucasians in the US donor registry dataset. For none of the tools there is a clear preference for the alleles, which are common in Caucasians. In fact, the typing accuracy was generally higher for the subset of alleles, which were common in other population groups.

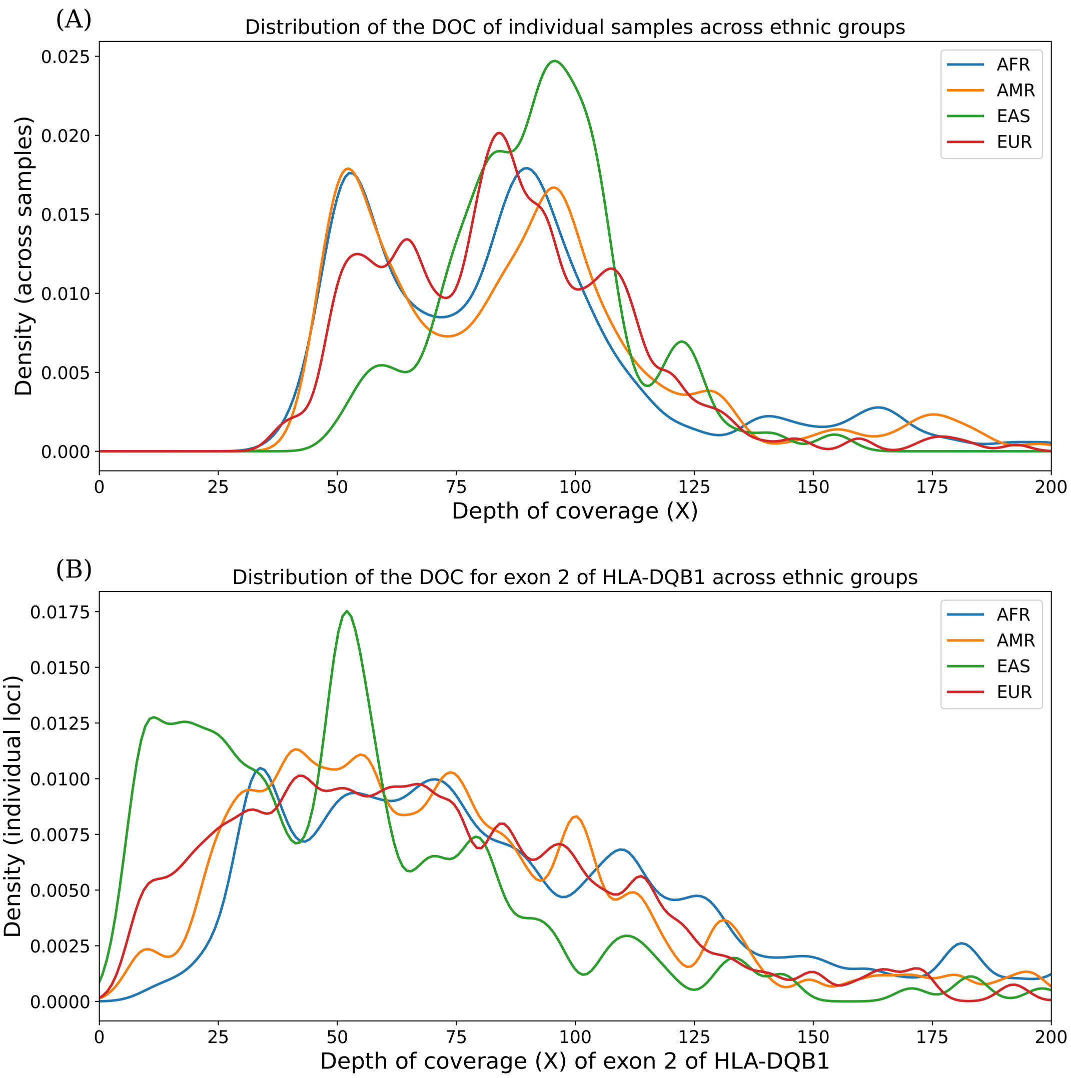

Figure S7. (A) The depth of coverage distribution for the four large ethnic groups in the 1000G dataset - Africans (AFR) Ad Mixed American (AMR) East Asian (EAS) and European (EUR). The few samples with very high depth of coverage (the largest with over 450X) is omitted here and the focus is instead on the coverage range that has the majority of the samples. The East Asian group has the highest mean (98.3X) and the highest median (93.1X) depth of coverage across the four ethnic groups. **(B)** The depth of coverage distribution specifically for the ARD coding exon (exon 2) of HLA-DQB1 stratified on the four population groups. The coverage was calculated by mapping the BAM files of the samples to the HLA-DQB1 sequences noted in the 1000G dataset. In cases of ambiguity in the 1000G dataset, the most frequent allele was chosen. In cases of ambiguity due to the typing resolution (e.g. an allele noted as HLA-A*01:01 in the 1000G dataset could be both HLA-A*01:01:01:01 and HLA-A*01:01:01:02), the allele with the lowest numerical name was chosen. Mapping was done using *BWA mem* v0.7.17 and the DOC was calculated using *mosdepth* (version 0.2.6). The precise genomic location of exon 2 varies between HLA-DQB1 alleles and the allele-specific exon 2 positions were therefore extracted from the IPD-IMGT/HLA database.

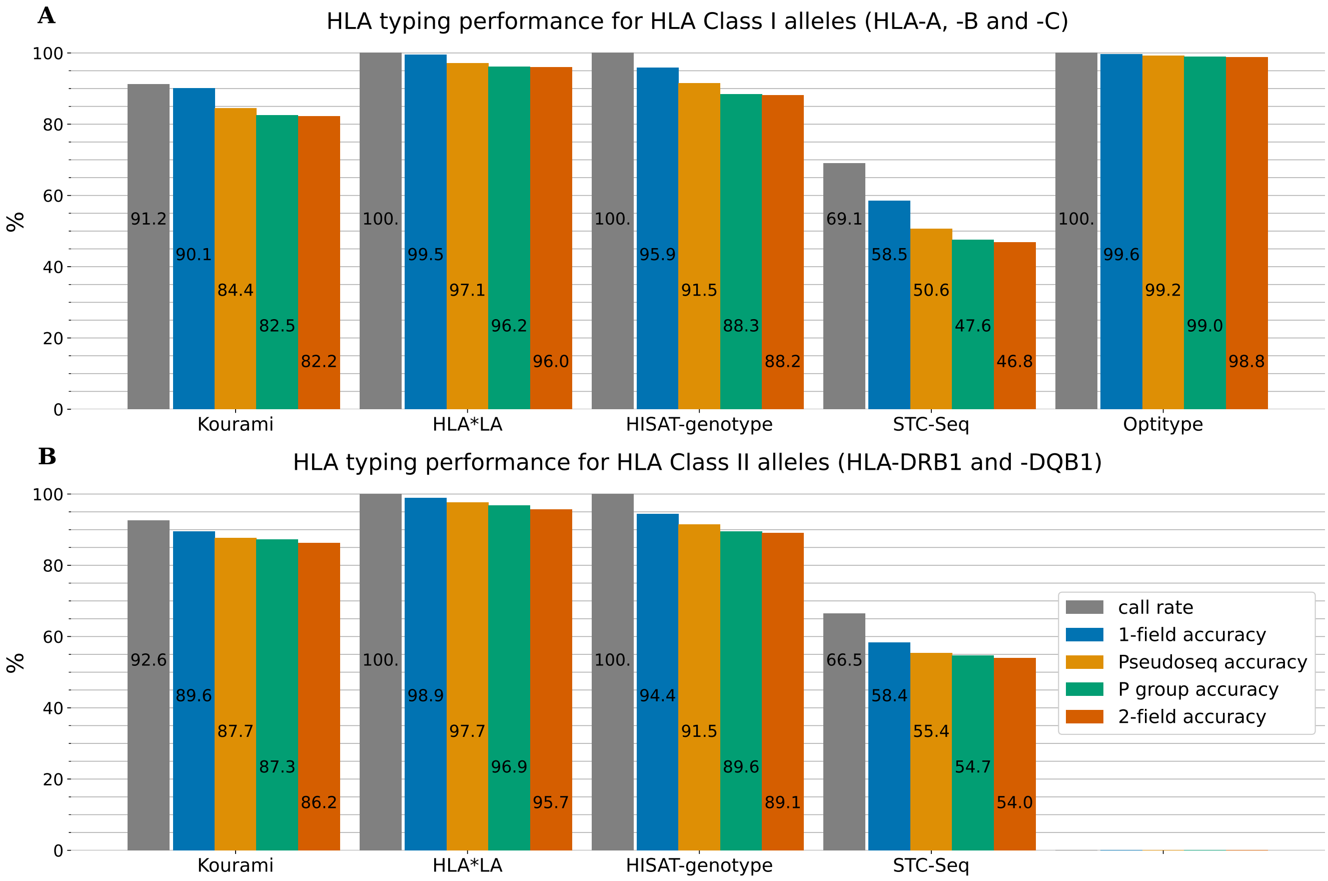

Figure S8. The performance of the HLA typing tools on a random subset containing half (414) of the 829 samples in the 1000 Genomes dataset used in this study. The tools show a similar performance for the 414 samples as for the 829 samples. This indicates that the 1000G dataset is large enough to give an accurate estimate of the tools' performances and that including more samples in the dataset likely would not yield notably different results.

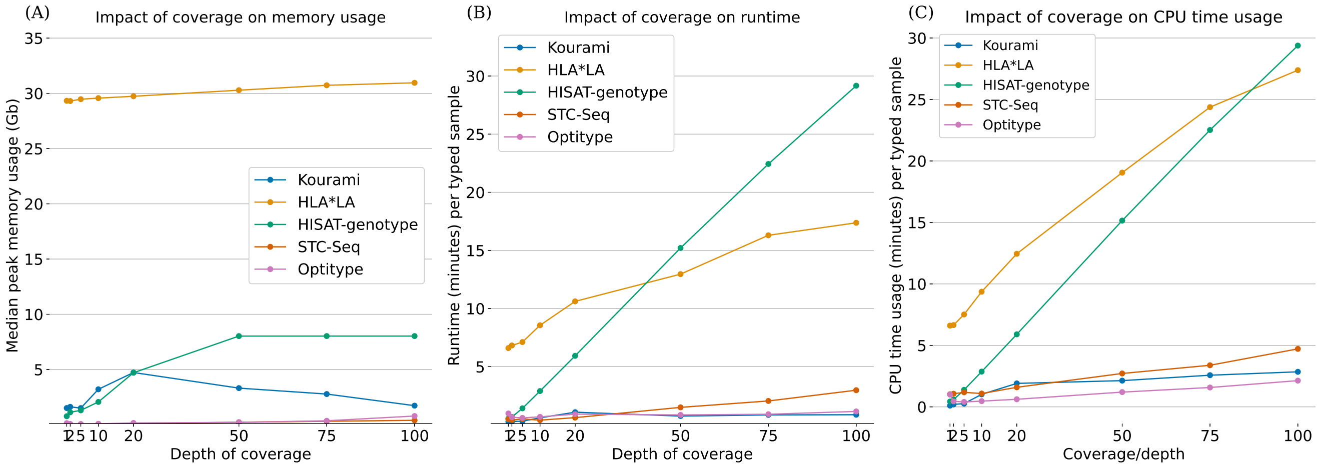

Figure S9. (A): An increased coverage generally increases the peak memory usage of the HLA typing tools, but not greatly. For HLA*LA there is almost no difference in peak memory usage for 1X and 100X. HISAT-genotype uses more memory when the coverage is increased, but only up until 8 GB. **(B)** and **(C)**: An increase in coverage also means that it takes more time for the tools to perform HLA typing. By extrapolating the trend for HISAT-genotype, the tool would need more than an hour in real time to type samples with a coverage of above 200X (using a setup identical to the one in this benchmarking study). Keep in mind, that these times are solely for the typing step and not the extraction of HLA reads, as illustrated in figure S1.

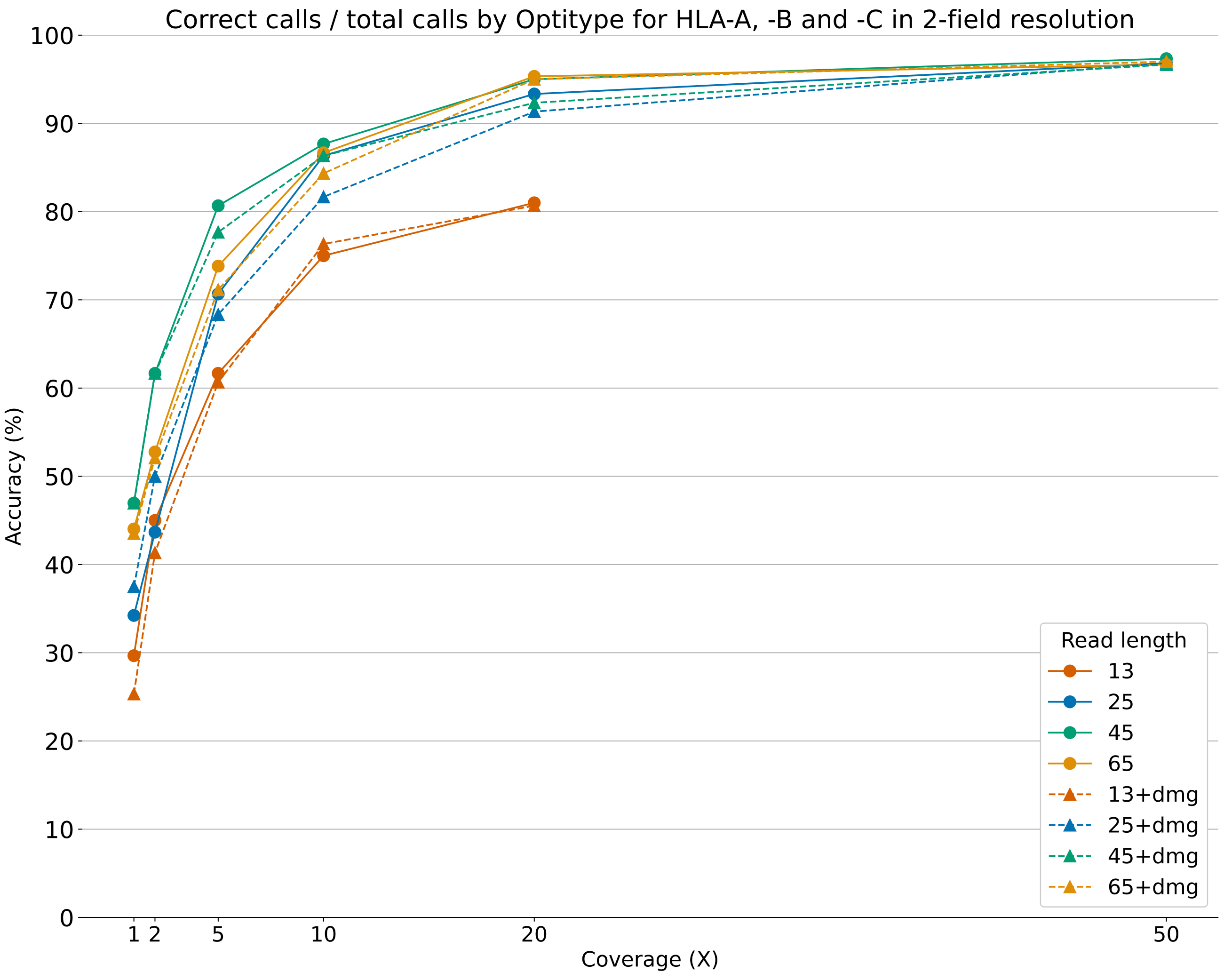

Figure S10. Optitype's call rate was not 100% for all combinations of read length, coverage and with/without read damage. This is either due to the fact that Optitype did not return a call *or* a sample did not have enough data to e.g. achieve a coverage of 50X, when the read length of each read was 13. This figure shows the amount of correct calls not out of the total calls but out of the calls that Optitype did make. The trend is very similar to the one shown in figure 5, but this figure shows how confident Optitype is on a call, when it *is* made.

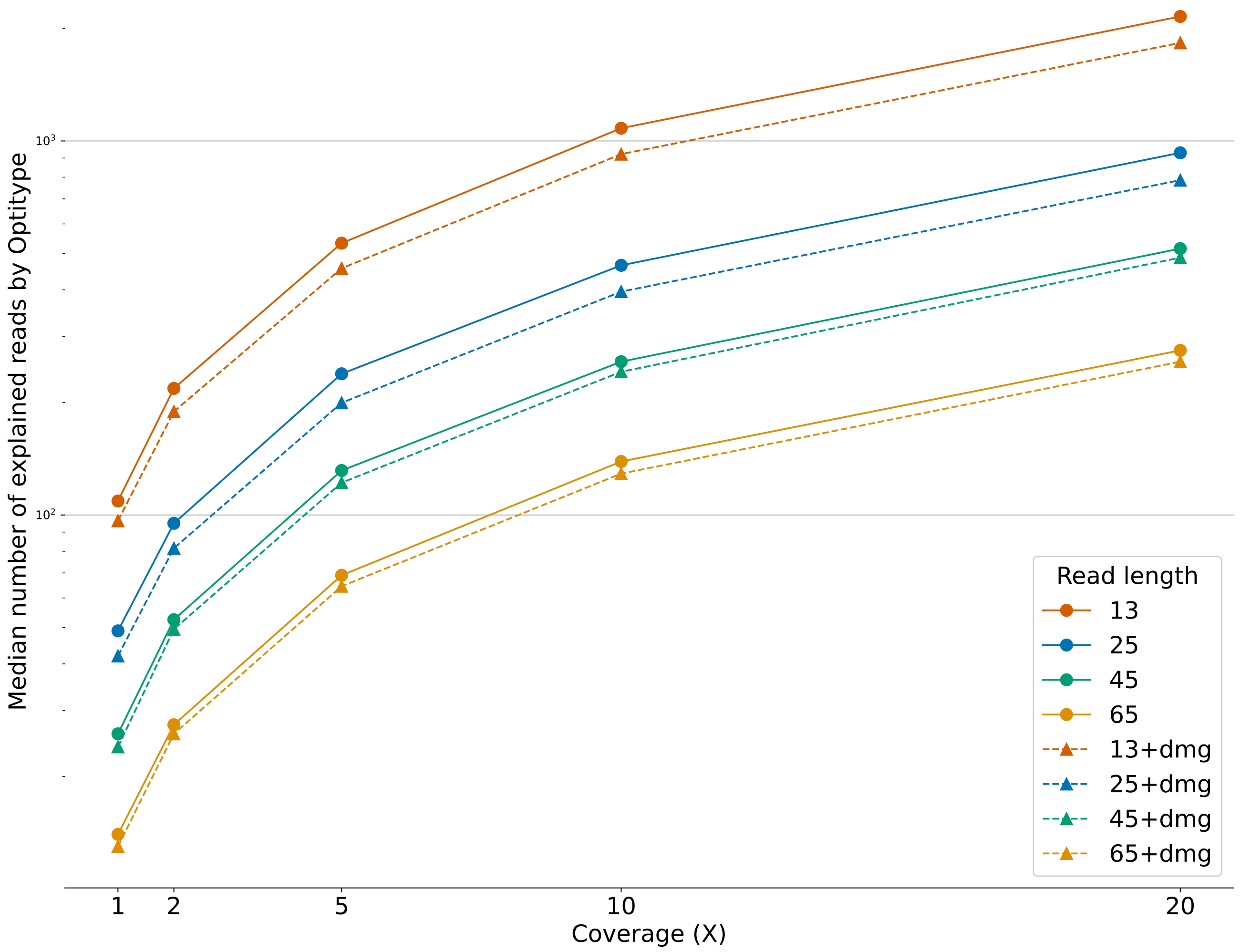

Figure S11. Optitype outputs the number of reads explained by its HLA allele prediction. The number of explained reads naturally depends on the coverage of the input sample. For a specific depth of coverage, there are more reads, if the read length is lower. This means, that there only are a few relevant HLA reads for samples samples with a read length of 65 and a coverage of 1X.

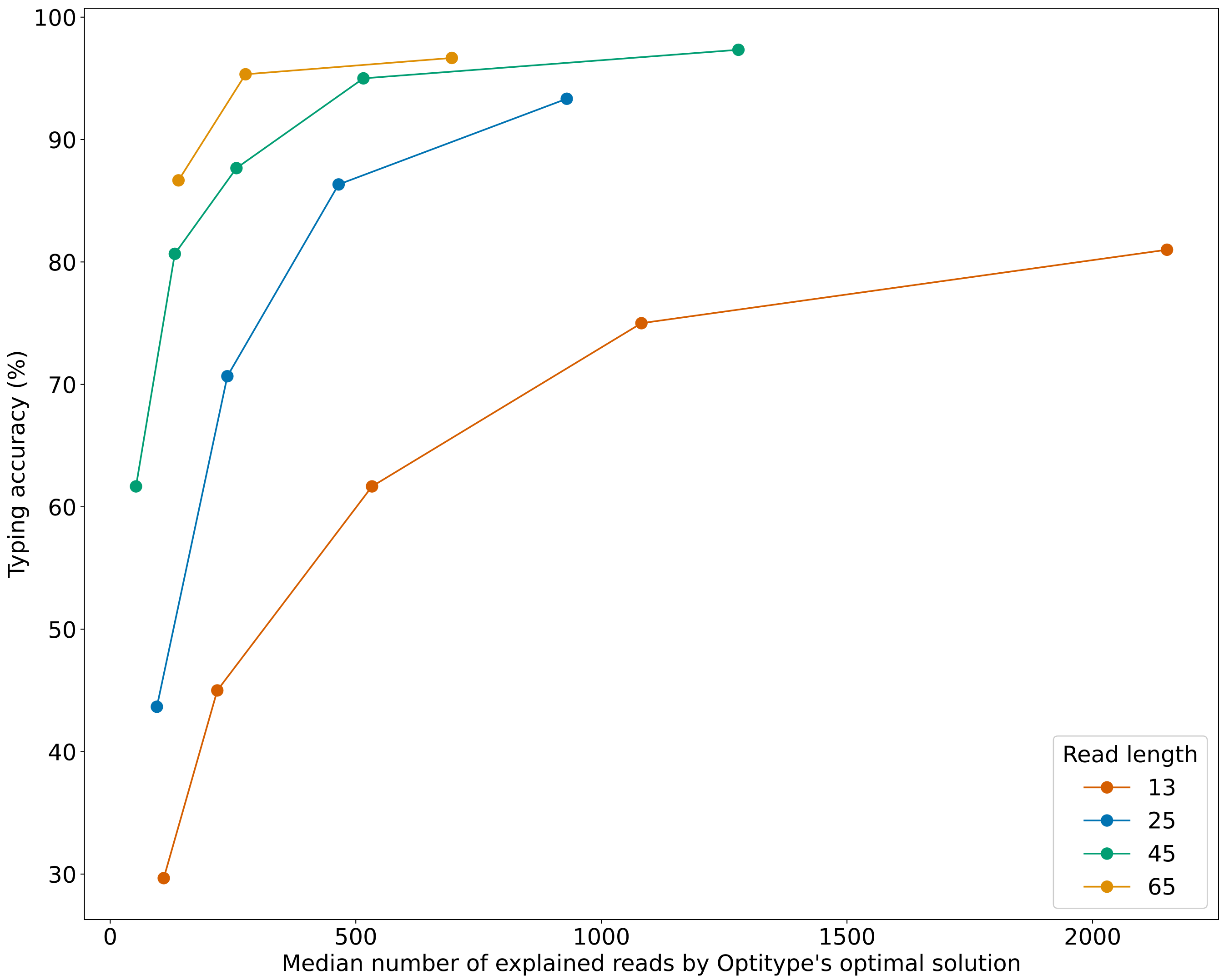

Figure S12. As the coverage and the number of explained reads by Optitype's optimal solution are highly correlated (see figure S11), the number of explained reads also correlate well with the typing accuracy. This figure only shows combinations of read length and coverage, where Optitype's call rate was 100%. Specifically, the depth coverages shown here are: 1X, 2X, 5X, 10X and 20X (for read length 13), 2X, 5X, 10X and 20X (for read length 20), 2X, 5X, 10X, 20X and 50X (for read length 45) and 10X, 20X and 50X (for read length 65).
